# Adenosine Receptor Profiling Reveals an Association between the Presence of Spare Receptors and Cardiovascular Disorders

**DOI:** 10.3390/ijms20235964

**Published:** 2019-11-27

**Authors:** Emmanuel Fenouillet, Giovanna Mottola, Nathalie Kipson, Franck Paganelli, Régis Guieu, Jean Ruf

**Affiliations:** 1 C2VN, INSERM, INRA, Aix-Marseille University, F-13015 Marseille, France; emmanuel.fenouillet@univ-amu.fr (E.F.); giovanna.mottola@univ-amu.fr (G.M.); nathalie.kipson@univ-amu.fr (N.K.); franck.paganelli@ap-hm.fr (F.P.); Jean.ruf@univ-amu.fr (J.R.); 2 INSB, CNRS, F-75016 Paris, France

**Keywords:** adenosine, adenosinergic system, cardiovascular disorders, coronary artery disease, receptor reserve, spare receptor, syncope

## Abstract

Adenosine and its receptors exert a potent control on the cardiovascular system. This review aims to present emerging experimental evidence supporting the existence and implication in cardiovascular disorders of specific adenosinergic pharmacological profiles, conforming to the concept of “receptor reserve”, also known as “spare receptors”. This kind of receptors allow agonists to achieve their maximal effect without occupying all of the relevant cell receptors. In the cardiovascular system, spare adenosine receptors appear to compensate for a low extracellular adenosine level and/or a low adenosine receptor number, such as in coronary artery disease or some kinds of neurocardiogenic syncopes. In both cases, the presence of spare receptors appears to be an attempt to overcome a weak interaction between adenosine and its receptors. The identification of adenosine spare receptors in cardiovascular disorders may be helpful for diagnostic purposes.

## 1. Adenosine and Its Receptors

A large literature reports that adenosine and its receptors (i.e., the adenosinergic system) exert many physiological functions, including a potent control of the cardiovascular system [1,2]. The adenosinergic system also plays a key role in cardiovascular pathophysiology, as adenosine receptors are expressed on most cell types in the cardiovascular system such as cardiomyocytes, endothelial cells, vascular smooth muscle cells, pericytes, and fibroblasts [1]. Adenosine receptors have been therefore evaluated as targets for the development of new therapeutic or diagnosis options for the cardiovascular system [2,3,4,5]. The goal of this review is to present experimental evidence supporting the existence and implication in cardiovascular disorders of specific adenosinergic pharmacological profiles agreeing with the concept of “receptor reserve” also known as “spare receptors” [6].

**Figure 1:**
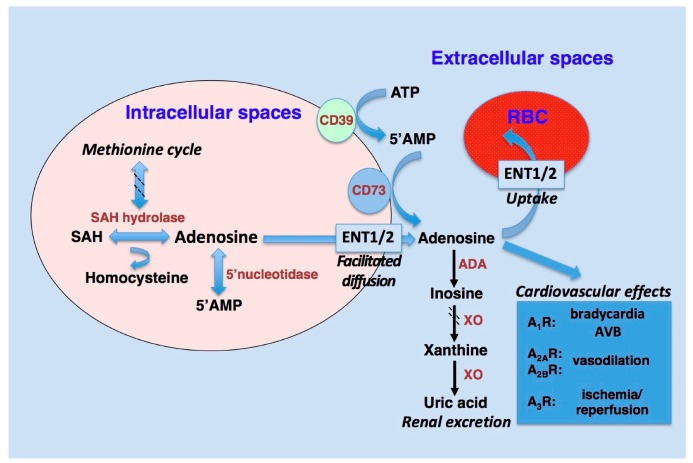
Adenosine metabolism and mechanisms. ADA: adenosine deaminase; AMP: adenosine monophosphate; ATP: adenosine triphosphate; AVB: atrioventricular block; ENT: equilibrative nucleoside transporter; SAH: S adenosyl-homocysteine; XO: xanthine oxidase; RBC: red blood cell.

In the extracellular space, adenosine is a ubiquitous nucleoside that derives mainly from the dephosphorylation of ATP into 5’AMP and of AMP into adenosine via cytosolic 5’-nucleotidase and extracellular CD39 and CD73, respectively [1] (Figure 1). In the cell, besides nucleotide dephosphorylation, adenosine comes from the methionine cycle through S-adenosylhomocysteine hydrolase and can undergo deamination to inosine or phosphorylation to AMP via adenosine deaminase (ADA) or adenosine kinase, respectively [1]. Under physiological conditions, adenosine concentrations are low. However, various situations including hypoxia or inflammation lead to a substantial increase of the nucleoside in interstitial spaces [7], which confers protection against tissue damage [8]. In the blood, adenosine concentration is mainly regulated via the equilibrative nucleoside transporters ENT1 and ENT2 of the erythrocytes [9] as well as via ADA that converts adenosine into non-toxic inosine and eventually to uric acid. ADA has a specific and key function within the adenosine pathway: While an acute increase in adenosine concentration is important to exert its functions, e.g., cardiovascular control, a persistently elevated adenosine concentration is detrimental for various cell populations [10]. For instance, high levels of adenosine lead to the accumulation of deoxyadenosine and deoxy-ATP, which are toxic for lymphocytes [10]. Thus, degradation mechanisms exist to offset the deleterious influences of chronically elevated adenosine levels in extracellular spaces.

Adenosine acts on target cells via four sub-types of G protein-coupled adenosine receptors (AR), namely, A_1_R, A_2A_R, A_2B_R, and A_3_R pending on their pharmacological properties and tissue distribution [1,2].

**Figure 2:**
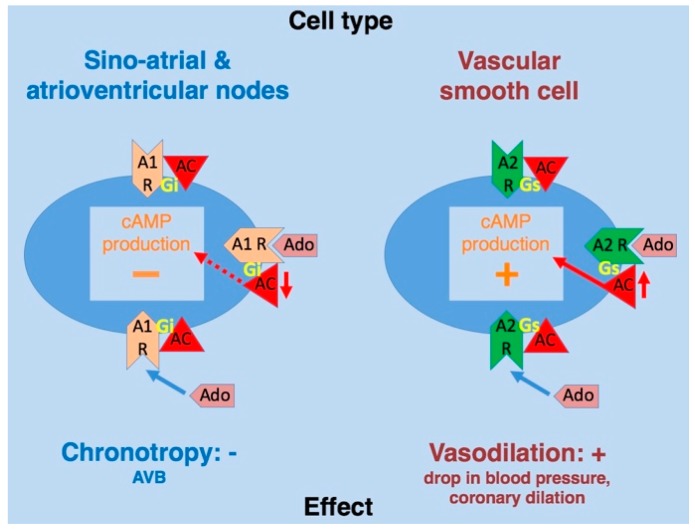
Adenosine receptor activation and cAMP production. The activation of effector cells by adenosine either increases (via A_2_ receptor) or decreases (via A_1_ receptor) cAMP production according to the cell type. Effects are indicated for illustration purposes (e.g., chronotropy, vasodilation). Ado: adenosine; A_1_R: A_1_ receptor; A_2_R: A_2_ receptor; AC: adenylylcyclase; Gs: G stimulating; Gi: G inhibiting; AVB: atrioventricular block. There is a direct relationship between cAMP production and biological effects.

A_1_R stimulation decreases cAMP production (Figure 2), which results in the inhibition of protein kinase A (PKA), activation of phospholipase C, and inhibition of voltage-dependent calcium channels [1]; activation of A_1_R also directly (cAMP-independent) activates the inwardly rectifying K^+^ current (I_KAdo_) [11]. Activation of A_2A_R inhibits voltage-gate Ca^++^ channels [12] and phosphorylates the cAMP-responsive element-binding protein, while A_2A/B_R activation increases cAMP production, PKA, and mitogen-activated protein kinases (MAPK). Finally, A_3_R stimulation decreases cAMP production and increases phospholipase C and MAPK phosphorylation [1]. An additional layer of complexity is brought by the findings that these receptors are engaged in homo- and hetero-oligomerization processes that result in specific functional properties [13,14].

## 2. Adenosine Receptors and the Cardiovascular System

Adenosine receptors are implicated in the regulation of heart rate and blood pressure [2], with adenosine release occurring in extracellular spaces in response to ischemia, hypoxia, and inflammation [7,15]. For instance, adenosine is released in the plasma by endothelial cells and myocytes during ischemia, particularly in the coronary artery: Its concentration in the coronary sinus is correlated with the extent of coronary artery stenosis. Accordingly, adenosine is considered a very early and sensitive marker of ischemia [16].

Adenosine exerts a variety of actions including adaptive responses in various physiological and pathophysiological situations via changes in adenosine production and tissue expression of AR subtypes [2,4]. Regarding the effects of A_1_R in supraventricular tissues, cAMP-independent activation—direct effects—of the I_KAdo_ current is the most important effect of adenosine [11]. Consequently, A_1_R activation leads to bradycardia or auriculo–ventricular block [17] via K^+^ and Ca^++^ current modulation [11]. In the cardiovascular system, activation of A_1_R and A_3_R also leads to protection against myocardium ischemia/reperfusion injuries and preconditioning [15].

Regarding A_2A_R, its activation leads to vasodilation via the cAMP pathway—indirect activation—and K_ATP_ channel opening in arterial smooth muscles [18]. Adenosine acts on coronary blood flow via A_2A_R and A_2B_R. Regarding A_2A_R, the adaptive response to an increase in di-oxygen needs during physical work is vasodilation, via an increase in adenosine plasma level (APL) that induces A_2A_R activation and cAMP production, cAMP production and coronary vasodilation being correlated [19]. A_2A_R activation also results in heart rhythm changes, since knock out (KO) mice for A_2A_R exhibit tachycardia, besides high blood pressure [20]. Additionally, activation of A_2A_R in coronary smooth muscle cells, endothelial cells, and mononuclear cells results in vasodilation [21], neo-angiogenesis, and increase in anti-inflammatory cytokines levels [1]. Coronary vasodilation is also triggered by ischemia in the myocardial cells via activation of A_2B_R [22,23]. Thus, activation of A_2A/B_R by high APL has short-term beneficial effects on the myocardium, although long-term A_2_R activation may be deleterious [1].

## 3. The Concept of Receptor Reserve and the Specific Case of Adenosine Receptors

Biological effects resulting from adenosine receptor activation are also submitted to inter-individual variations that have been attributed to particular pharmacological characteristics such as the receptor reserve mechanism described thereafter. The receptor reserve concept was originally defined as the fraction of receptors not required to achieve maximal response by a full agonist [6,24]. In this context, T-cell receptors are the archetype of the spare receptor model, because the excess of receptors on peripheral T cells is required for responses to ligands of variable affinities and low concentrations of agonists [25]. Thereby, the spare receptor model can be designed as a signal amplification system in which the effectiveness of the response to different ligands, full or partial agonists and possibly inverse agonist, can be quite complex due to the mixing of the effects of partial activation of receptors with those of signal transduction [26].

From a pharmacological point of view, experimental findings show that correlations between receptor density, ligand affinity, and tissue response may strongly vary depending on physiological and pathophysiological situations, tissue origin, and type of agonist [27]. Apparently inconsistent conclusions also emerge from observations that, on some occasions, a maximum biological effect can be achieved via binding of an agonist to only a small proportion of its receptors on target cells [28]. To address such apparent discrepancies between common ligand-binding models based on the original receptor occupancy theory proposed by Clark in the 1930s—that is, the more receptors are activated, the higher the response [29]—and a variety of experimental observations that the pharmacological response of a given agonist may not be proportional to the fraction of bound receptors, a concept was developed and named receptor reserve or spare receptor [6,21,28]. This concept that dissociates the occurrence or intensity of a biological response from the number of ligand-bound receptors has at least one implication: a maximal response can be achieved when only a low fraction of the receptors is occupied by an agonist [6,21,28].

From a biochemical point of view, the presence of spare receptors is characterized by a high K_D_/EC_50_ ratio, with maximal biological effects (evaluated via EC_50_, i.e., the ligand concentration leading to half maximal effect) resulting from binding to only a sub-fraction of receptors (evaluated via the dissociation constant K_D_, i.e., the concentration of ligand at which 50% of receptors are occupied at equilibrium). This biochemical definition of the spare receptor concept, i.e., the EC_50_ is lower than the K_D_ value, is consistent with the pharmacological definition of a receptor reserve, which implies that maximal effect is obtained while many surface receptors are unoccupied by the ligand.

In summary, a receptor reserve depends on the intrinsic efficacy of a given agonist to activate a receptor as well as on the density of receptors and the efficiency of the signaling pathway of a given tissue. A receptor reserve enables responses that are rapid, transient, and sensitive to low agonist concentration and/or low-affinity binding. In other words, the presence of the spare receptor mechanism is expected to provide a high-efficiency mechanism.

## 4. Experimental Strategies to Address the Adenosinergic System in Cardiovascular Tissues

Experimental strategies that have been employed to identify the presence of a receptor reserve have included the evaluation of responses to agonists following the inactivation of a subset of receptors using an irreversible antagonist. When a receptor reserve is available, a partial agonist can behave as a full agonist [21,30,31]. Partial agonists are therefore potential tools to detect the presence of spare receptors. More recently, the use of tetracycline-inducible cell lines to control the amount of M1 muscarinic acetylcholine receptors showed that the activity of an allosteric agonist is dependent on receptor reserve [32]. Besides the use of irreversible organic ligands, alternative approaches to evidence spare receptors were sought, and in particular, the presence of spare A_2A_R was detected via the use of an anti-A_2A_R antibody that possesses agonist properties, called Adonis [33,34,35,36]. 

Adonis is a monoclonal IgM antibody that binds with high affinity a linear epitope localized in the second extracellular loop of the human A_2A_R [37]. This IgM behaves, during the course of binding experiments, as an irreversible ligand of A_2A_R [35] and triggers cAMP production following A_2A_R binding [33,34,35,36]. On the basis of these properties, experimental procedures were designed, as reported elsewhere [33,34,35,36], to determine both binding (K_D_) and functional (EC_50_ regarding cAMP production) parameters of the mAb–A_2A_R binding reaction. In some pathophysiological conditions, Adonis binding to A_2A_R was found to occur with a high K_D_/EC_50_ ratio [35,36], indicating the presence of spare A_2A_R. These observations made Adonis an original tool to detect the presence of spare A_2A_R. It was further developed as a potential diagnostic/screening marker, as discussed thereafter.

Besides the use of a ligand with specific pharmacological properties to detect spare A_2A_R, an additional point makes it feasible to indirectly evaluate an A_2A_R reserve associated with cardiovascular tissues, which otherwise would require difficult, highly invasive sampling procedures, despite the strong A_2A_R expression in the coronary system. Indeed, the properties of A_2A_R expressed by peripheral blood mononuclear cells (PBMC) mirror the properties of its counterpart produced by cardiovascular tissues: (i) The expression of A_2A_R by PBMC reflects A_2A_R expression by the left ventricle in cardiac transplant recipient [38]; (ii) The expression levels of A_2A_R in PBMC and in coronary and aortic tissues were found to be correlated in coronary artery disease (CAD) patients [39]; (iii) A_2A_R expression evaluated on PBMCs correlates with A_2A_R expression evaluated in femoral artery tissues in patients with lower extremity peripheral artery disease (LE-PAD) [40]; (iv) A_2A_R expressed in PBMC and aortic tissues of CAD patients displayed a similar capacity to induce cAMP production following agonist stimulation, and hence are similarly functional [40]. These data support the conclusion that A_2A_R expression and function are submitted to systemic regulation, probably because PBMC are exposed to the blood flow, communicate with coronary tissues via the lymphatic network, and consequently are submitted to adenosinergic stress as the heart and cardiovascular tissues. That expression and function of A_2A_R in cardiovascular tissues correlate with expression and function of A_2A_R in PBMC provides a unique opportunity to address the adenosinergic system and its behavior in ischemic conditions in the coronary arteries.

## 5. Adenosine Receptor Reserve and the Cardiovascular System

Some information is already available regarding the presence and role of adenosine receptor reserves in the cardiovascular system. Following binding to A_1_R on guinea pig atrial myocytes, adenosine regulates the atrial rhythm by activating inwardly rectifying K^+^ current with half maximal I_KAdo_ activation, requiring 40% of A_1_R occupancy [41,42]. In strong contrast, A_1_R activation inhibits isoproterenol-stimulated L-type Ca^++^ current, with half maximal activation achieved when only 4% of A_1_R are occupied, which supports the presence of a receptor reserve [41].

As to adenosine binding to A_2_R, the EC_50_ value for coronary vasodilation is much lower than the EC_50_ value necessary to influence the heart rhythm in guinea pig [21], suggesting that signal transduction is much more efficient to achieve coronary vasodilation than chonotropic or dromotopic inhibition, with activation of only 5% of A_2A_R leading to half maximal coronary conductance and vasodilation [21]. These results are consistent with the presence of spare A_2A_R. A pharmacological and therapeutic consequence may be that antagonists that recognize A_2A_R with an affinity in the range of adenosine require higher concentration to displace adenosine from A_2A_R when a large proportion of unoccupied receptors exists. Additional data will be presented below to highlight a role for spare A_2A_R in cardiovascular disorders such as CAD and neurocardiogenic syncopes (NCS).

Finally, and besides its effect on heart rhythm and blood flow, adenosine also interacts with the cardiovascular system via blood and, hence, PBMCs: The upregulation of A_2A_R results in adenosinergic T-cell immunosuppression during hypoxia [43], and conversely, down-regulation of A_2A_R in PBMC of CAD patients probably promotes inflammation [44]. This hypothesis is consistent with the observation that activation of A_2A_R on CD4^+^ T-cells inhibits inflammation and reduces the size of myocardial infarction [45]. The expression levels of these receptors and their functional activity are therefore of paramount importance in coronary blood flow maintenance but also in inflammation.

### 5.1. Spare A_2A_ Receptors in Coronary Artery Disease

CAD occupies an important place in cardiovascular diseases and is estimated to be responsible, according to the World Health Organization, for nearly 7 million deaths a year, or 12.8% of all deaths [46]. CAD encompasses various disorders resulting from insufficient di-oxygen supply to the myocardium and ranging from transient ischemia to myocardial necrosis [47]. In healthy subjects, adenosine and A_2_R adapt coronary vasodilation to cardiac di-oxygen demand following muscle exercise and hence heart work such as during the exercise stress test (EST); the test result is “negative” when there are no chest pain and/or T segment (ST) depression on the ECG at peak exercise. In CAD and/or cardiac failure, the vasodilator response to myocardial hypoxia/ischemia appears to be generally unable to correct the myocardial ischemia that is produced during EST, myocardial di-oxygen delivery being unable to match myocardial di-oxygen consumption due to altered coronary blood flow (the test result is “positive” when there are chest pain and/or ST depression at peak exercise) [47].

**Figure 3:**
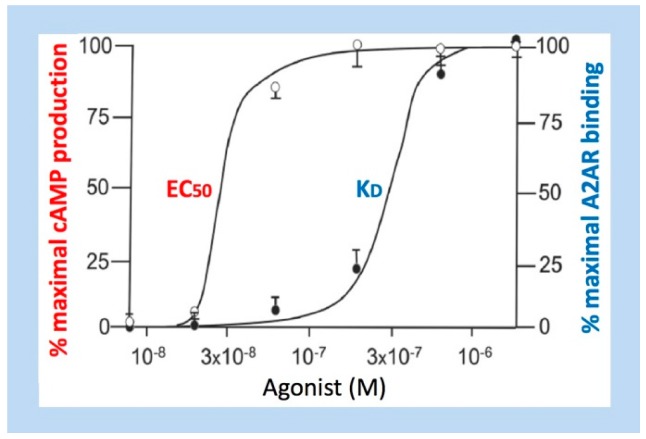
Relationship between EC_50_ and K_D_ regarding A_2A_R expressed in the lymphocytes of a coronary artery disease patient. Dose–response curves resulting from binding of an agonist to A_2A_R expressed on the surface of lymphocytes isolated from a patient with ischemia induced by the exercise stress test (EST). White circles: cAMP production. Black circles: agonist binding. The fact that EC_50_ < < K_D_ indicates the presence of a receptor reserve (after ref. [35], with modifications).

In CAD patients where the coronary blood flow is reduced, a decrease in A_2A_R production is observed in coronary tissues [39,48]. Such a decrease has been attributed to receptor export via extracellular vesicles in a context of high homocysteine plasma level [49]. Furthermore, low APL is observed in CAD at rest [48,50]. At rest, CAD patients have therefore both low APL [48,50] and low A_2A_R expression [36,48]. During positive EST, however, APL strongly increases in patients with positive EST, which probably results from the myocardial ischemia process that occurs during the test in a context of obstructive CAD [35]. This result is consistent with the observation that during experimental coronary reactive hyperemia, adenosine increases coronary blood flow up to three-fold in a dose-dependent manner [22]. Regarding A_2A_R expression, patients with positive EST exhibit lower receptor production than patients with negative EST [35]. Besides abnormal low A_2A_R production and low APL, the presence of spare receptors is detected in CAD patients with either positive EST [35] or positive fractional flow reserve (FFR), which can accurately measure blood pressure and flow through a specific part of the coronary arteries [36], i.e., two inducible ischemia conditions. While the K_D_ of the A_2A_R agonist used is similar in patients and controls, the EC_50_ value (related to the biological effect triggered by the agonist and monitored here via cAMP production) is lower in patients. More importantly, while EC_50_ is greater than K_D_ in healthy subjects, the reverse situation occurs in patients, EC_50_ being significantly lower than K_D_ in the vast majority of patients with positive EST [35]. The conclusion is that a high K_D_/EC_50_ ratio, which is consistent with the presence of spare receptors, is associated with signs of myocardial ischemia (Figure 3).

In CAD patients, the K_D_/EC_50_ positive threshold is estimated to be close to 2 for significant ischemia, as documented by FFR [36]. At this point, 50% of free receptors remain. Thus, for a K_D_/EC_50_ > 2, there is a reserve of receptors > 50%, which may be associated with myocardial ischemia [36].

These results also suggest that in CAD, the presence of spare A_2A_R is an adaptive response to inducible ischemia. Finally, low A_2A_R expression was also reported in LE-PAD patients [40]. In this population, spare A_2A_R were found only when LE-PAD was associated with inducible myocardial ischemia [40]. Together, these results support a specific association between A_2A_R with properties of spare receptor and myocardial ischemia. Whether the presence of spare A_2A_R in CAD patients is an adaptive response to chronic myocardial ischemia or results from genetic predisposition needs further investigations.

### 5.2. The Functional Paradox of Spare Receptor in CAD

**Figure 4:**
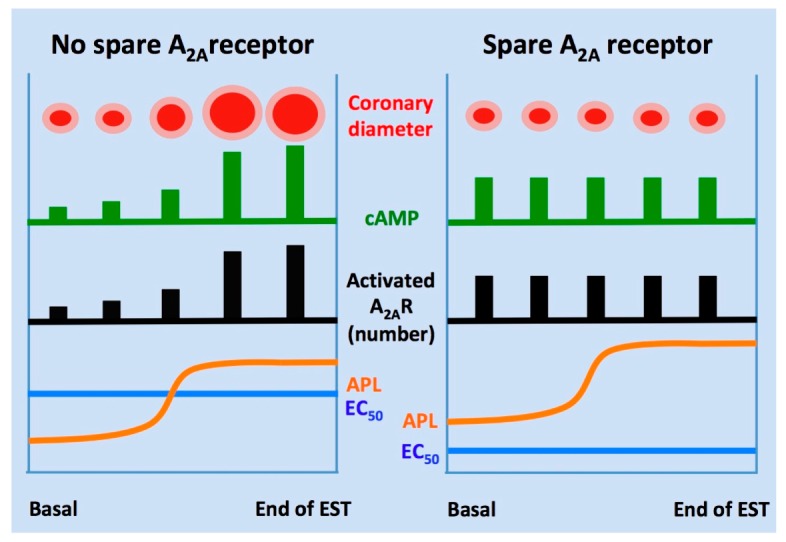
Spare A_2A_ receptors and coronary artery disease. During the EST, the adenosine plasma level (APL) increases to improve di-oxygen supply to the myocardium by increasing coronary artery diameter, and hence blood flow. In the absence of spare A_2A_ receptors, increase in APL leads to coronary vasodilation via A_2A_ receptor activation and cAMP production, cAMP production and vasodilation being correlated. In the presence of spare A_2A_ receptors, the production of cAMP in basal conditions is already maximal and cannot be further increased when di-oxygen supply needs to be increased during EST, because APL level is much above the EC_50_ value: no significant coronary vasodilation occurs.

The results reported above highlight an apparent paradox associated with the presence of spare A_2A_R in CAD (Figure 4). In CAD patients with inducible ischemia objectified by a positive EST, however, the strong increase in APL during EST fails to adjust coronary vasodilation to workload [35]: the presence of spare A_2A_R is therefore not sufficient to provide efficient vasodilation during exercise in a context of low A_2A_R expression level and despite a significant increase in APL. In contrast, in CAD patients without sign of inducible ischemia (negative EST or flow fraction reserve), it can be considered that the regulation of coronary vasodilation does not imply spare A_2A_R, since normal receptor expression and increased adenosine production are sufficient to accommodate the increased workload [35]. An additional paradox of the CAD situation is that spare A_2A_R in CAD are detected in a context of low receptor expression, contrary to the report of Shryock and Belardinelli [21], using an animal model where a receptor reserve was identified in a context of high receptor expression. To explain this apparent discrepancy, it can be hypothesized that A_2A_R is engaged in monomers or oligomers [14]. In the situation where oligomerization occurs, a single site occupied within the A_2A_R oligomer results in maximal effects according to the revisited spare receptor theory, whereas, in the context of chronic disease such as CAD, too few oligomerized A_2A_R are expressed to produce effective vasodilation, even in the presence of high agonist plasma levels [51]. This mechanism may also explain why the reserve increases (K_D_/EC_50_ > 2) in a context of low A_2A_R expression and high order of oligomerization.

### 5.3. Spare A_2A_ Receptors in NCS

NCS are frequent in the general population (1–3%) and severely alter the quality of life of patients. About 3–5% of emergency hospital admissions, 1–2% of hospitalizations, and 0.28% of mortality rate are attributable to NCS [52]. NCS is characterized by a loss of consciousness associated with a loss of postural tone due to a drop in blood pressure, usually preceded by prodromes like nausea, sweat, or abdominal pain. In some cases, there are no prodromes, and the loss of consciousness occurs roughly [17,53,54]. For diagnostic purposes, NCS can be reproduced using the head-up tilt test as well as via exogenous administration of adenosine, which demonstrates that the adenosinergic system is implicated in NCS [55,56]. In NCS, in general, the loss of consciousness is attributed to a strong vasoplegia via A_2A_R activation or to bradycardia or auriculo–ventricular block via the activation of A_1_R [17,53]. Both mechanisms may also occur.

Adenosinergic system profiling highlights an association between APL and unexplained syncope in patients without prodromes, carotid sinus syncope, and vasovagal syncope vs. normal control subjects [53]. The clinical impact of adenosine depends on its concentration, adenosine receptor expression level, and the presence of receptor reserve [33,34]. In syncope without prodromes where APL values are very low (“sudden syncope”) [53,54,55]—and are even mainly below the EC_50_ value for A_1_R—even a modest acute increase in APL in a context of A_1_R reserve may activate a sufficient number of A_1_R located within the sinus node and in the atrioventricular node, their activation resulting in atrioventricular block. In contrast, in vasovagal syncope, the high APL levels are compatible with the activation of low-affinity A_2A_R located in the vessels and producing vasodilation and desensitization of high-affinity A_1_R [53,54,55]. These results are consistent with the report that these patients also show a high incidence of positive tilt tests, where a strong increase in APL is observed [53,54,55,56].

**Figure 5:**
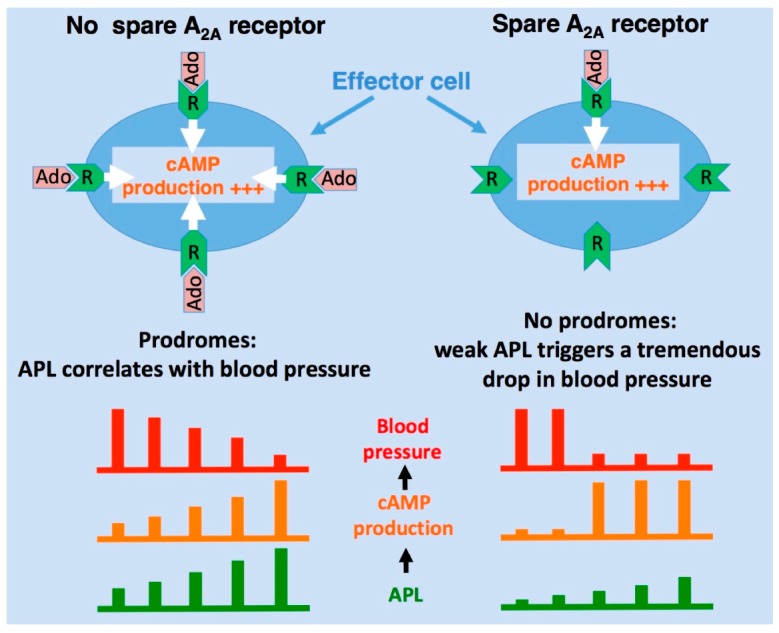
Spare A_2A_ receptors and neurocardiogenic syncopes. In the absence of spare A_2A_ receptors, the activation of target cells by adenosine leads to cAMP production and biological effects that are proportional to adenosine plasma levels (APL; left panel). In the presence of spare A_2A_ receptors, a weak increase in APL leads to a dramatic drop in blood pressure due to strong and rapid vasodilation, auriculo–ventricular block, or both (right panel).

The presence of spare A_2A_R was first found in patients with NCS but not in healthy subjects [33,34], which raises the question of the role of spare A_2A_R in the pathophysiology of NCS subtypes, especially in the group without prodromes and with low APL. The presence of spare receptors probably precipitates vasoplegia and/or atrioventricular block in conditions where APL exceeds the EC_50_ value (Figure 5). This hypothesis is consistent with the fast drop in systolic blood pressure and the absence of prodromes observed in sudden syncope. 

In summary, the presence of spare A_1_R and/or A_2A_R may explain inter-individual distinct responses following APL increase, resulting from either exogenous adenosine administration or endogenous adenosine production during the head-up tilt test. In addition, particular purinergic profiles, which may be genetically predetermined [56], are associated with different common forms of neutrally mediated syncope that can be classified as low-, normal-, and high-adenosine syncope [53]. In this context, the presence of spare adenosine receptors can explain the strong inter-individual susceptibility to an increase in exogenous or endogenous adenosine and the sharp drop in blood pressure observed during the NCS episode.

## 6. Spare A_2A_R as a Screening Tool

The data presented above show that spare A_2A_R can be readily detected in PBMC of patients. The predictive value of EST for detecting patients with CAD is limited, and a reliable method for the diagnosis of minimal cardiac ischemia, such as biomarkers, is therefore highly desired to avoid the use of EST and FFR. The development of a non-invasive procedure that can specifically detect the presence of spare A_2A_R on PBMC is therefore desirable to screen patients with CAD and at high risk of myocardial ischemia [35,36].

Regarding NCS, a classification based on adenosinergic profiling was recently proposed [53]: The syncope with prodromes (vasovagal syncope) is associated with high APL and high A_2A_R expression on PBMC, a specific single-nucleotide polymorphism in *A_2A_R* gene, and a positive head-up tilt test [56], while the sudden syncope without prodromes is associated with low APL levels and low A_2A_R expression [53,54,55]. In the latter case, the administration of exogenous adenosine causes a tremendous drop in blood pressure [55], which further supports the presence of spare A_2A_R (Figure 5). Thus, the detection of spare A_2A_R may help to screen NCS patients.

## 7. Conclusions

According to the original concept of receptor reserve, the specific advantage conferred by a receptor reserve-like response is that an excess of receptors extends the spectrum of ligands or concentrations a system can deal with. In cardiovascular disorders, spare adenosine receptors seem to have emerged to compensate for a low extracellular adenosine level and/or a low adenosine receptor number, such as in syncope without prodromes [17,53,54,55] or CAD [35,36,39,48]. In both cases, the presence of spare receptors is an attempt to overcome conditions of weak interaction between adenosine and its receptors. Whether the presence of spare receptors results from a genetic predisposition or an acquired phenomenon remains unknown. Finally, this review provides strong evidence that the identification of adenosine spare receptors in CAD, NCS, and probably, yet unexplored cardiovascular disorders may be helpful in patient screening for diagnostic purposes.
